# Host‐associated chemical cues mediating host‐finding behaviour in the larval ectoparasitoid *Cephalonomia tarsalis*

**DOI:** 10.1002/ps.70896

**Published:** 2026-05-10

**Authors:** Lidia del Arco, Benjamin Fürstenau, Cristina Castañé, Jordi Riudavets

**Affiliations:** ^1^ IRTA, Sustainable Plant Protection Cabrils (Barcelona) Spain; ^2^ Julius Kühn Institute Federal Research Centre for Cultivated Plants Institute for Ecological Chemistry, Plant Analysis and Stored Product Protection Berlin Germany

**Keywords:** biological control, olfactometer assays, sawtoothed grain beetle, semiochemicals, stored product pests

## Abstract

**BACKGROUND:**

Understanding how parasitoids locate their hosts is essential for improving the effectiveness of these insects as biological control agents. The bethylid ectoparasitoid *Cephalonomia tarsalis* is a key natural enemy of the sawtoothed grain beetle *Oryzaephilus surinamensis*, an important stored product pest. While some aspects of its host‐finding behaviour are understood, the significance of larval host‐associated volatile cues remains largely unclear. This study investigated the influence of host‐specific odours on host location by *C. tarsalis* females.

**RESULTS:**

Volatile compounds released from fourth‐instar larval faeces were identified by gas chromatography–mass spectrometry and their behavioural relevance was evaluated using Y‐tube olfactometer bioassays alongside odours from different host stages (live larvae and adults) and from host‐associated food substrates. Females responded positively to most of the tested odours, including those from host adults, larval faeces, and host‐associated food substrates. Among the compounds identified in the faecal volatiles of host larvae, 1‐pentadecene was found to be significantly attractive. In flight‐cage experiments, 1‐pentadecene induced behavioural responses in test parasitoids that were similar to those elicited by larval faeces.

**CONCLUSION:**

Host‐associated volatile cues, particularly those derived from larval faeces, play a key role in mediating the host‐searching behaviour of *C. tarsalis* females. These findings provide a basis for the development of semiochemical‐based approaches to enhance the efficiency of this biological control agent for stored‐product pests. © 2026 The Author(s). *Pest Management Science* published by John Wiley & Sons Ltd on behalf of Society of Chemical Industry.

## Graphical abstract

Host‐associated volatiles mediate host location in *Cephalonomia tarsalis*, with faecal odours from fourth‐instar larvae, particularly 1‐pentadecene, attracting females and supporting the biological control of stored‐product pest beetles.

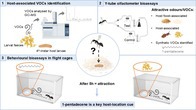

## 1 INTRODUCTION

In view of the growing demand for resilient and sustainable agriculture and the rising risk of pests becoming resistant to commonly used pesticides, traditional chemical control is increasingly viewed and discussed critically by society and policymakers. Biorational approaches have become a valuable alternative to chemical pesticides within integrated pest management strategies, as they effectively control pest infestations while minimising adverse impacts on human health and the environment. One such approach is biological control, which relies on the use of living organisms to suppress or reduce pest populations in response to infestations or as a preventive measure to maintain pest densities below established damage thresholds.^1^

Hymenopteran parasitoids are effective biological control agents due to their high host specificity and ability to substantially reduce pest populations, even at low host densities.^2^ Parasitoids rely on various sensory signals to detect host habitats and locate suitable hosts, with chemical cues playing a particularly important role by guiding them in this process. These cues may originate from one or multiple sources and are central to the host‐finding process. Successful orientation requires parasitoids to recognise host‐specific odours within a complex chemical environment and to discriminate these informative signals from unspecific background noise.^3^, ^4^ Potential odour sources include host‐derived products such as faeces, silk, chemical trails and pheromones, as well as volatile compounds released from the host's food substrate.^5^, ^6^, ^7^

Although parasitoids have proven to be effective biological control agents in agricultural and horticultural systems, their adoption for managing stored product pests in warehouses and similar facilities remains limited. This is due to several factors, including advances in physical control methods, as well as higher costs, slower action, and the still existing efficacy gap relative to conventional pesticides. Nevertheless, beneficial arthropods offer promising potential for broader use in stored product pest management, particularly as knowledge of their biology and chemical ecology continues to advance. In this context, the specific role of semiochemicals in odour‐mediated host‐searching behaviour remains poorly understood for many species and may narrow their effective implementation. Such knowledge is crucial for broadening our understanding of their chemical communication and host‐location mechanisms and, ultimately, for improving the practical application of parasitoids in biological control programs in storage facilities.^8^ To date, research in this area has primarily focused on species belonging to the families Pteromalidae, Braconidae, Ichneumonidae and Bethylidae.^9^ For instance, behavioural experiments on the chemically mediated host‐finding process of the bethylid wasp *Holepyris sylvanidis* (Bréthes) (Hymenoptera: Bethylidae) have shown that the application of synthetic host‐specific volatile compounds increases the host‐searching activity and parasitisation rates in females.^10^ These findings open up new avenues in the field of biologically based stored product protection. Further investigation and transfer to other parasitoid–host systems is required.

The ectoparasitoid *Cephalonomia tarsalis* (Ashmead) (Hymenoptera: Bethylidae) specifically targets the larvae and pupae of the sawtoothed grain beetle *Oryzaephilus surinamensis* (L.) (Coleoptera: Silvanidae).^11^ This beetle is one of the most destructive pests of stored grain products. It feeds on kernels that have been broken or damaged by other pests, causing substantial quantitative and qualitative losses.^12^, ^13^, ^14^ Like many other parasitoids, *C. tarsalis* uses chemical cues to locate its host. Females initially orient towards long‐range attractive volatile compounds emitted by substrates associated with the host habitat, such as healthy and damaged grains.^15^ Once within the host habitat, females primarily rely on medium‐range attractants, including odours released from host residues such as host faeces, which guide them to areas infested with their hosts and facilitate accurate localisation of larvae.^11^, ^15^, ^16^, ^17^ At closer range, short‐range chemical signals deposited along larval trails enable females to locate hosts within the infested substrate.^5^, ^11^, ^15^ On direct contact with a larva, cuticular chemical cues, particularly long‐chain hydrocarbons, together with larval movement detected through antennal exploration, determine host recognition and acceptance by the parasitoid, whereas dead larvae are rejected.^11^, ^15^ Once accepted, the female paralyses the larva by stinging it with her ovipositor, injecting venom.^11^ However, while short‐range cues such as cuticular hydrocarbons are known to mediate host recognition on contact in *C. tarsalis*, the identity and behavioural relevance of host‐associated volatile compounds involved in medium‐ and long‐range host location remain unclear. In particular, it is unknown which host‐associated volatile compounds, including those derived from larval faeces, guide females towards their hosts prior to contact, or how these compounds influence host‐searching behaviour. These knowledge gaps highlight the need for further research into the role of semiochemicals in host‐searching parasitoids. Identifying behaviourally relevant host‐associated volatiles could provide tools to enhance the efficiency and reliability of parasitoids in the management of stored‐product pests by enabling the application of attractant odours in practical settings.

The present study aimed to assess the influence of host‐specific odours on the host‐finding behaviour of the larval ectoparasitoid *C. tarsalis*. To this end, three specific objectives were addressed. First, host‐associated volatile compounds released from the faeces of fourth‐instar *O. surinamensis* larvae were identified using gas chromatography–mass spectrometry (GC–MS). Second, the behavioural responses of *C. tarsalis* females to host‐specific odours were evaluated using Y‐tube olfactometer bioassays, including volatiles emitted by host‐associated food substrates, different host instars, and selected synthetic compounds previously identified from larval faecal volatiles. Finally, the effect of a candidate attractive compound, identified from larval faeces, was assessed on the parasitoid host‐searching behaviour conducted under larger‐scale experimental conditions in a flight‐cage experiment.

## 2 METHODS AND MATERIAL

### 2.1 Insect colonies

The stock colonies of *O. surinamensis* and *C. tarsalis* were established using individuals collected from warehouses of different cereal commodities in northeast Spain. *Oryzaephilus surinamensis* was reared on a diet consisting of a mixture of whole‐wheat flour and brown rice (*Oryza sativa* L. var. *japonica*). *Cephalonomia tarsalis* was reared on fourth‐instar larvae of *O. surinamensis*, and females aged 1–5 days were used in all bioassays. The stock colonies were maintained under environmentally controlled conditions (28 ± 2 °C, 70 ± 2% relative humidity (RH), 16 h:8 h light:dark).

### 2.2 Collection and analysis of volatiles from the host faeces and the rearing substrate

To obtain samples of *O. surinamensis* larval faeces, the adults were first transferred to a glass jar (13.5 cm high × 9 cm internal diameter) containing 300 g of brown rice to produce a cohort of fourth‐instar larvae. Once the larvae reached the fourth instar, they were collected from the rearing jars and transferred to an empty stainless‐steel dish. After defecating in the dish for 4 h, the faeces were collected and stored in a glass vial at −20 °C until used. On average, approximately 16.5 mg of faeces was produced daily by 200 larvae.

Volatile organic compounds (VOCs) emitted by the faeces of fourth‐instar *O. surinamensis* larvae and from uninfested brown rice (rearing substrate) were collected by dynamic headspace sampling. Brown rice was chosen because parasitoids showed no response in a preliminary olfactometer test, indicating that it does not emit attractive VOCs. Comparison of the chemical profiles of the two sources enabled the identification of compounds produced by the host itself, rather than unmetabolized derivates of the food substrate.

Charcoal‐filtered air (150 mL min^−1^) was passed over each sample (*N* = 3), placed in a 100‐mL Erlenmeyer flask. A faecal sample of 0.75 g, the minimum amount required to ensure sufficient volatile collection, and 5 g of uninfested brown rice were used per sample. Volatiles were trapped for 24 h at room temperature on Tenax TA (60/80 mg) desorption tubes (6 cm long × 6.0 mm outer diameter; Gerstel, Mühlheim, Germany). A blank sample was included in each volatile collection.

The headspace volatiles were analysed by GC–MS using a 7890A gas chromatograph coupled with a 5975C VC quadrupole mass spectrometer (Agilent Technologies, Waldbronn, Germany). The trapped VOCs were desorbed from the filter in a thermal desorption unit (TDU) (Gerstel, Mülheim, Germany). To start, the TDU was held at 23 °C for 1 min, then heated at a rate of 720 °C min^−1^ to 250 °C, where it was held for 3 min. Subsequently, the VOCs were concentrated in a programmable temperature vaporization‐type inlet (CIS 4; Gerstel) at −150 °C. The temperature of the cryotrap was then increased to 250 °C at a rate of 12 °C s^−1^ to transfer the VOCs to the column. The VOCs were transferred in splitless mode to a DB‐5MS UI capillary column (30 m × 0.25 mm × 0.50 μm; J & W Scientific) with helium as a carrier gas at a flow rate of 1.2 mL min^−1^. The temperature programming of the oven included three stages: initially set at 45 °C and held for 2 min, the temperature increased at a rate of 3 °C min^−1^ to 105 °C without any holding period, followed by an increase rate of 1 °C min^−1^ until 111 °C and finally temperature increased at a rate of 3 °C min^−1^ up to 210 °C (held for 27 min). Mass spectra of the VOCs were obtained by electron impact ionization at 70 eV with a source temperature of 250 °C (scan mode range 35–350 m/z). Standard compounds were dissolved in hexane and transferred to an empty desorption tube. They were injected separately with a solvent delay of 3.5 min, using the GC conditions described above. Ten microlitres of a 10 ng μL^−1^ methylnonanoate solution (Sigma–Aldrich) were injected as internal standard (IS). Individual faecal and rice volatiles were quantified relative to the peak area of the IS. Samples were standardised by calculating the mean amount of each compound (in nanograms per gram of sample). Retention indices (RI) were calculated^18^ using a standard hydrocarbon mixture (*n*‐C7–C40; Sigma Aldrich, Taufkirchen, Germany) containing odd‐ and even‐numbered *n*‐alkanes. Volatile compounds were tentatively identified by comparing their mass spectra and RI values with those of authentic standards and/or entries in the NIST Mass Spectral Library and published reference data.^19^, ^20^

### 2.3 Y‐tube olfactometer bioassays

The olfactory responses of *C. tarsalis* females to host‐associated odours were evaluated using a Y‐tube glass olfactometer. The olfactometer comprised a main tube (21 cm long × 15 mm internal diameter) with two 15‐cm long arms set at an angle of 135° to each other. Each arm was connected by polytetrafluoroethylene tubing to a 50‐mL glass flask, one containing the test sample and the other serving as a blank sample. Charcoal‐filtered air was circulated through the sample flasks, the arms, and the main entrance of the Y‐tube at a rate of 100 mL min^−1^. The Y‐tube was placed on a table at a 45° angle and uniformly illuminated by a dimmed neon office lamp (30 lx) situated 50 cm below the olfactometer. All bioassays were conducted under similar room conditions from 9:00 to 17:00 h at 21 ± 1 °C. Prior to the bioassays, the Y‐tube olfactometers, flasks, and connection tubes were cleaned with detergent, rinsed with distilled water and 70% ethanol solution, and dried in an oven at 155 °C for 4 h. Parasitoid females were acclimatised in the experimental room for 1 h before the bioassay.

Based on our GC–MS analyses, compounds uniquely detected in host faecal volatiles and not in the brown rice substrate were considered for subsequent behavioural tests. Of these, those previously reported as faecal volatiles of other stored product pest beetle species^21^, ^22^, ^23^ or as attractants of bethylid parasitoids^23^ were prioritised, and a subset of compounds representing different chemical classes was selected for bioassays. These compounds were (*E*)‐2‐octenal (94%), (*E*)‐2‐nonenal (97%), octanoic acid (98%), and phenylacetaldehyde (90%) (all purchased from Sigma‐Aldrich), as well as 1‐pentadecene (95%) (obtained from TCI Europe). For all bioassays, each compound was dissolved in 10 μL of hexane at the target concentration and applied to a 4‐cm diameter piece of filter paper.

Preliminary olfactometer tests were conducted using solely 1‐pentadecene and (*E*)‐2‐nonenal to determine the effective concentration range, as higher concentrations may have a repellent effect.^24^ Both compounds were evaluated at three concentrations against the solvent (hexane): 1‐pentadecene at 10.20, 1.02, and 0.10 ng μL^−1^, and (*E*)‐2‐nonenal at 10.65, 1.06, and 0.10 ng μL^−1^. Parasitoids responded (moved towards either the compound or the blank arm) only at the lowest concentration (0.10 ng μL^−1^) of both compounds. At higher concentrations, response rates were very low (≥75% non‐responders), with most test females remaining at the release point (not moving up the olfactometer tube and thus making no choice between test and control), resulting in an insufficient number of responding individuals for meaningful statistical analysis. These data were therefore not included in the statistical analyses. Based on these results, a concentration of 0.10 ng μL^−1^, corresponding to a total applied dose of 1 ng, was used in the subsequent olfactometer bioassays. Table 1 summarises the Y‐tube olfactometer bioassays conducted to evaluate the attraction of *C. tarsalis* females to odours emitted by the host and its food sources.

**Table 1:** Y‐tube olfactometer bioassays assessing the attraction of *Cephalonomia tarsalis* females to host food‐associated odours, host‐derived odours, and selected synthetic compounds identified by gas chromatography–mass spectrometry from *Oryzaephilus surinamensis* larval faeces (*N* = 30 adult females)

Odour or volatile compound tested	Comparison	Amount
Host food‐associated odours	Non‐infested paddy rice *vs* air	5.0 g
	Non‐infested rearing diet *vs* air	5.0 g
Host‐associated odours	Larval faeces *vs* air	0.75 g
	Adults *vs* air	10 individuals
	Larvae *vs* air	10 individuals
Synthetic volatile organic compounds identified from larval faeces	1‐pentadecene *vs* hexane	1.0 ng
	(*E*)‐2‐nonenal *vs* hexane	1.0 ng
	Octanoic acid *vs* hexane	1.0 ng
	Phenylacetaldehyde *vs* hexane	1.0 ng
	(*E*)‐2‐octenal *vs* hexane	1.0 ng
Attractive synthetic compound *vs* natural faecal source	1‐pentadecene *vs* larval faeces	1.0 ng *vs* 0.75 g
	1‐pentadecene *vs* larval faeces	1.0 ng *vs* 0.10 g

All synthetic compounds were evaluated at 0.10 ng μL^−1^ in a total volume of 10 μL.

Each bioassay consisted of releasing a female parasitoid at the entrance of the main arm, which was immediately covered with a mesh (to avoid escape), and its response observed for a maximum of 300 s, with a different female used in each replicate. A positive response was recorded when the parasitoid moved at least 3 cm beyond the intersection of one of the two arms and did not return within 10 s. Once a positive response was recorded, or when the maximum observation time had elapsed without a response, the parasitoid was removed and the assay concluded. Each odour or VOC was tested until a response was obtained from 30 parasitoids, except for odours where the response rate did not exceed 30% of the total parasitoids tested. Every five tests, the Y‐olfactometer tube was replaced and the position of the odour sources was alternated to avoid positional bias. The different treatments were conducted in blocks of 15 tests, and their order randomly varied to minimise potential sequence effects. Additionally, when testing the VOCs, the loaded filter papers were replaced with new ones after each run to account for evaporation. Odour sources associated with host individuals and host food sources were replaced at 30‐min intervals. A blank control using no odour (air *vs* air) was performed to ensure the absence of side preference and, consequently, to eliminate any positional effects from the setup. Parasitoid females that did not respond (remained at the entrance of the olfactometer and did not move up the main tube or not choose either arm) were excluded from the statistical analysis.

### 2.4 Behavioural bioassays in flight cages

A flight cage experiment (32.5 × 32.5 × 77.0 cm) was conducted to assess the effects of 1‐pentadecene and *O. surinamensis* larval faeces on the host‐finding effectiveness of *C. tarsalis* females. The olfactory source was placed in a Petri dish (3.5 cm diameter) at the centre of an adhesive trap (12 × 12 cm) on one side of the cage. At the opposite side of the cage, at a distance of 70 cm from the olfactory source, five females of *C. tarsalis* were released. The number of *C. tarsalis* stuck on the trap was counted after 1, 2, 3, 4, 5, and 24 h. Subsequently, all parasitoids, the adhesive trap, and the Petri dishes containing the olfactory sources were removed and the cages were cleaned with 70% ethanol. A control treatment, identical in setup but with the Petri dish containing no olfactory source, was included to establish a baseline for the likelihood of parasitoids being captured in the trap by chance.

The experiment was conducted in a dark room maintained at 28 ± 1 °C and 33 ± 7% RH, and six flight cages were placed in two rows of three cages, each located adjacent to one another, with 1.10 m between the rows. One row contained cages with the olfactory source, while the other row contained cages with no olfactory source. An oscillating table fan (14.4 × 8.0 × 18.8 cm, 5000 mAh; Comlife, China) was placed in front of each row, on the side of the cages containing the olfactory source to ensure a smooth airflow towards the parasitoid release point. The bioassay was repeated three times, resulting in nine replicates (cages) for each olfactory source and its respective control treatment. The following odours and VOCs were tested: (i) 0.10 g of fourth‐instar host larval faeces, (ii) 10 μL of 1‐pentadecene (1.2 ng μL^−1^), (iii) 0.10 g of fourth‐instar host larval faeces + 5 g of paddy rice, and (iv) 10 μL of 1‐pentadecene (1.2 ng μL^−1^) + 5 g of paddy rice. To compensate for greater dilution in the larger assay arena, 1‐pentadecene was applied at a higher concentration than that used in the olfactometer, providing a total of 12 ng per cage. Paddy rice was used this time because it is the most common form of rice storage in warehouses.

### 2.5 Data analysis

Differences in parasitoid preference for the odours or VOCs offered in the Y‐tube olfactometer assays were analysed by two‐sided binomial tests. The null hypothesis was that the proportion of individuals choosing either arm was equal.

In the behavioural bioassays conducted in flight cages, the number of *C. tarsalis* captured per hour in traps for each control treatment was analysed using a generalised linear model (GLM) fitted with a Poisson distribution (link = log). Since no significant differences were observed among the control treatments, they were grouped into a single control treatment by calculating the mean captures per hour. This grouped control treatment was subsequently included as one of the five treatments tested.

To quantify overall parasitoid attraction over time, the area under the curve (AUC) was calculated for each treatment (control, larval faeces, 1‐pentadecene, larval faeces + paddy rice, and 1‐pentadecene + paddy rice), with cumulative captures as the response variable and the hour of sampling as the independent variable. The resulting AUC values were used as the response variable in a one‐way ANOVA to test for differences among treatments, followed by Tukey's *post hoc* tests. Then, the number of *C. tarsalis* captured in the five treatments and the capture rates per hour based on treatment and experimental time were analysed using a GLM fitted with a quasi‐Poisson distribution (link = log) to account for overdispersion. *Post hoc* comparisons were conducted using Tukey's adjustment for multiple comparisons. For all statistical analyses, a nominal significance of 5% (*P* < 0.05) was applied. All statistical analyses were conducted using RStudio software.^25^

## 3 RESULTS

### 3.1 Identification of host faeces volatiles

**Figure 1:**
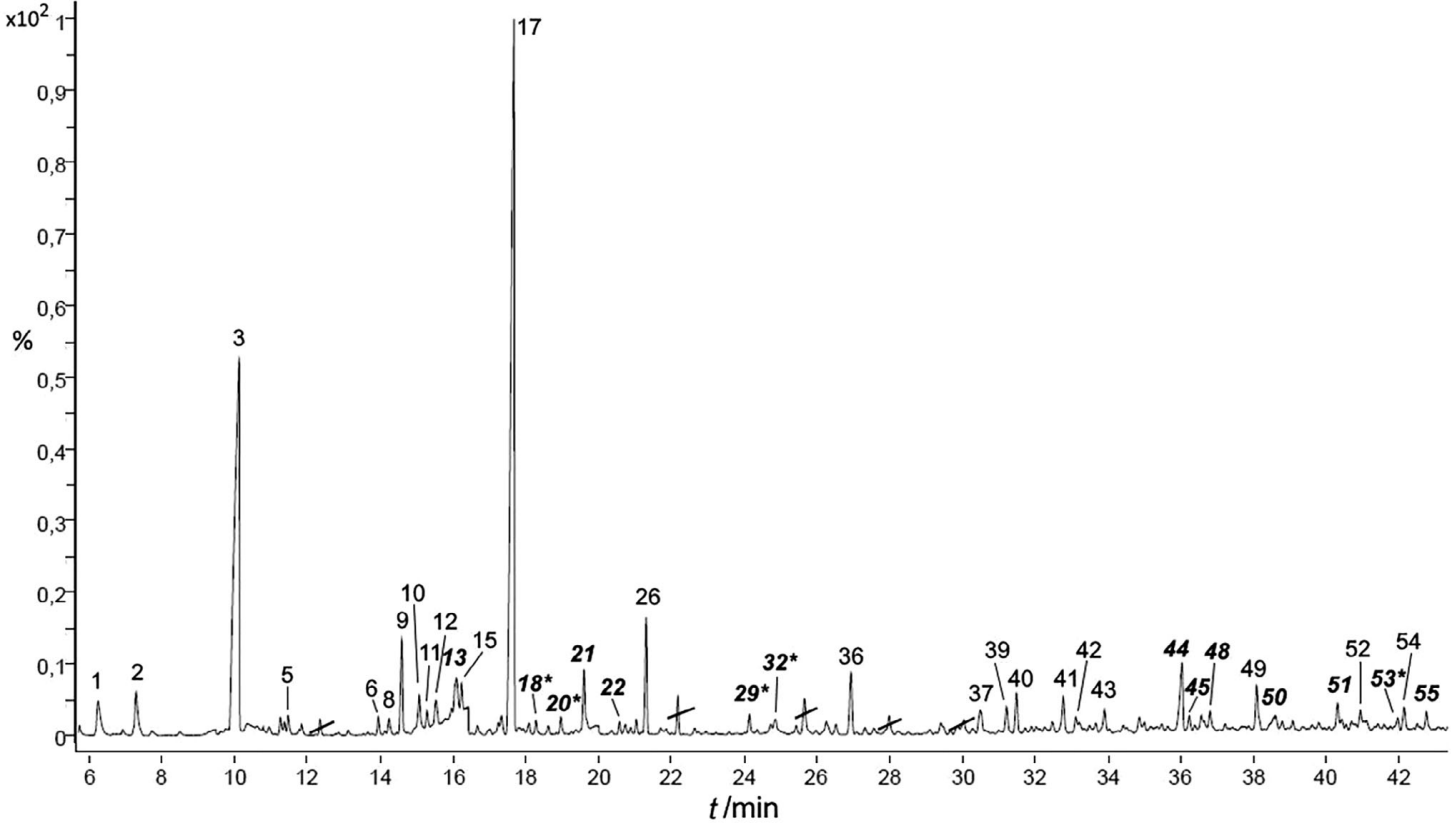
Partial gas chromatography–mass spectrometry profiles of headspace volatiles of 0.75 g of fresh faeces of *Oryzaephilus surinamensis* larvae collected for 24 h and separated on a DB‐5MS column. Reproducible peaks are indicated by numbers above peaks corresponding to compounds listed in Table 1. Numbers in bold and italics represent unique compounds only present in host‐associated odour collected from fresh larval faeces. Numbers with asterisks were tested in Y‐tube olfactometer bioassays. Dashed peaks are contaminations/artifacts.

In total, 55 VOCs were identified from fresh faeces of fourth‐instar *O. surinamensis* larvae and from non‐infested brown rice used to rear them (Table 2 and Fig. 1). When qualitatively comparing faecal and non‐infested rice odours, 24 VOCs were shared between the two samples, 14 VOCs were detected exclusively in the headspace of fresh larval faeces (highlighted in bold in Table 2), and 17 VOCs were absent from the faecal odour but present in the non‐infested rice odour.

**Table 2:** Mean amounts (ng) of volatile organic compounds (± standard error) detected in headspace extracts of fresh host larval faeces and uninfested brown rice (0.75 and 5 g, respectively) collected for 24 h (*N* = 3)

No.	Compound	RI_calc_ ^†^	RI_lit_ ^‡^	Host larval faeces (ng)	Non‐infested rice (ng)
1	1‐Pentanol	771	766	30.5 ± 5.8	31.4 ± 18.6
2	Hexanal	800	805	39.4 ± 2.8	128.2 ± 11.0
3	1‐Hexanol	870	869	442.3 ± 50.5	182.3 ± 25.9
4	Nonane	900	899	0	13.0 ± 5.4
5	Heptanal	902	900	10.7 ± 3.6	11.4 ± 1.0
6	(*E*)‐2‐Heptenal	956	955	11.9 ± 2.2	15.8 ± 2.1
7	1‐Ethyl‐3‐methyl‐benzene	960	964	0	15.1 ± 5.9
8	Benzaldehyde	963	964	15.7 ± 6.8	15.8 ± 23.8
9	1‐Heptanol	970	970	47.6 ± 5.2	42.2 ± 2.9
10	1‐Octen‐3‐ol	980	980	38.6 ± 7.9	25.2 ± 3.8
11	6‐Methyl‐5‐hepten‐2‐one	985	986	8.6 ± 1.4	15.9 ± 3.6
12	2‐Pentyl‐furan	990	992	15.2 ± 3.5	10.6 ± 4.9
**13**	**Hexanoic acid**	**998**	**997**	**22.2 ± 9.5**	0
14	Decane	1001	999	0	83.2 ± 6.2
15	Octanal	1004	1004	49.5 ± 43.3	57.9 ± 14.8
16	2‐Ethyl‐1‐hexanol	1030	1029	0	5.4 ± 10.9
17	D‐Limonene	1032	1031	350.9 ± 200.5	13.6 ± 2.6
**18**	**Phenylacetaldehyde** *	**1045**	**1043**	**9.1 ± 1.7**	0
19	5‐Ethyldihydro‐2(3H)‐furanone	1052	1055	0	12.9 ± 2.2
**20**	**(*E*)‐2‐Octenal** *	**1059**	**1056**	**8.8 ± 0.8**	0
**21**	**1‐Octanol**	**1071**	**1072**	**39.9 ± 1.5**	0
**22**	**Fenchone**	**1091**	**1094**	**7.4 ± 1.3**	0
23	2‐Nonanone	1091	1091	0	10.0 ± 0.8
24	Unknown	1095		0	21.4 ± 3.7
25	Undecane	1100	1101	0	15.6 ± 7.8
26	Nonanal	1104	1105	77.8 ± 15.6	149.2 ± 17.9
27	Unknown	1116	1122	0	38.1 ± 0.8
28	5‐Methyl‐undecane	1151	1154	0	15.1 ± 0.1
**29**	**(*E*)‐2‐Nonenal** *	**1157**	**1155**	**11.2 ± 0.7**	0
30	2‐Methyl‐undecane	1161	1163	0	29.1 ± 1.3
31	3‐Methyl‐undecane	1168	1169	0	22.6 ± 0.5
**32**	**Octanoic acid** *	**1170**	**1172**	**12.9 ± 1.9**	0
33	Unknown	1185		0	37.5 ± 13.8
34	2‐Decanone	1191	1192	0	8.5 ± 0.3
35	Dodecane	1201	1199	0	114.8 ± 6.9
36	Decanal	1207	1209	30.2 ± 24.7	35.6 ± 3.1
37	2‐Methyl‐1‐decanol	1264		17.2 ± 2.4	34.0 ± 2.9
38	2,10‐Dimethyl‐undecane	1266	1265	0	16. ± 1.6
39	Unknown	1275		30.6 ± 12.0	23.8 ± 3.7
**40**	**Unknown**	**1280**		**18.9 ± 4.9**	0
41	Tridecane	1300	1299	27.4 ± 1.4	76.8 ± 7.1
42	Unknown	1307	1285	4.3 ± 1.7	22.5 ± 1.6
43	Unknown	1321		34.3 ± 17.1	28.3 ± 2.4
44	Dihydro‐5‐pentyl‐2(3H)‐furanone	1360	1361	45.7 ± 9.3	24.5 ± 4.6
**45**	**Unknown**	**1365**		**9.1 ± 0.6**	0
46	2‐Undecenal	1366	1368	0	13.2 ± 1.6
47	Unknown	1372		12.8 ± 2.8	10.9 ± 1.8
**48**	**2,6,10‐Trimethyl‐dodecane**	**1376**	**1376**	**39.4 ± 21.9**	0
49	Tetradecane	1400	1400	45.1 ± 9.2	24.6 ± 3.6
**50**	**Dodecanal**	**1410**	**1409**	**23.6 ± 4.7**	0
**51**	**(*E*)‐6,10‐dimethyl‐5,9‐Undecadien‐2‐one**	**1448**	**1448**	**24.1 ± 4.9**	0
52	unknown	1461		18.2 ± 11.6	12.8 ± 1.8
**53**	^§^ **1‐Pentadecene** *	**1483**	**1489**	**12.2 ± 2.0**	0
54	unknown	1487		19.8 ± 3.1	11.0 ± 2.0
**55**	**Pentadecane**	**1500**	**1500**	**18.0 ± 5.0**	0

VOCs exclusively detected on host‐associated fresh larval faeces are highlighted in bold. IS, methylnonanoate (100 ng).

* Compounds tested in Y‐tube olfactmeter bioassays.

^†^ Calculated retention index (RI_calc_.) on a DB‐5MS (30 m × 0.25 mm internal diameter × 0.25 μm) fused silica capillary column.

^‡^ Retention index of standards and/or references (RI_lit_.) on a DB‐5MS fused silica capillary column or similar.

^§^ Compounds elicited behavioural responses by female parasitoids.

### 3.2 Y‐tube olfactometer bioassays

**Figure 2:**
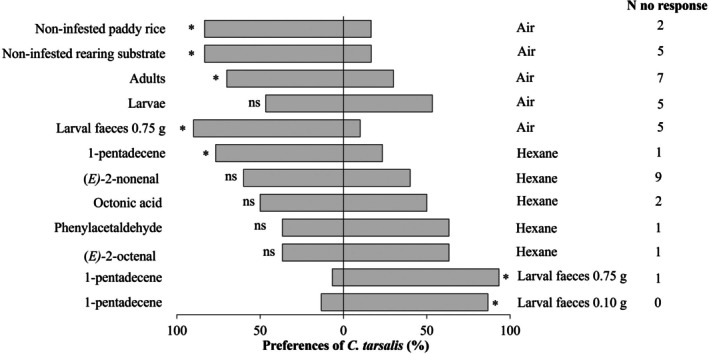
Behavioural responses of *Cephalonomia tarsalis* to host‐associated odours and synthetic volatile organic compounds in pairwise Y‐tube olfactometer tests (*N* = 30 per comparison). Asterisks indicate significant differences in the parasitoid preferences (*P* < 0.05); ns indicates no significant differences in the parasitoid preferences. For details of odour treatments, selected synthetic compounds, and experimental comparisons, see Table 1.

*Cephalonomia tarsalis* females responded positively to nearly all odours from host food sources and host individuals tested, consistently choosing the test over the control arm (Fig. 2). Among host food‐associated odours, non‐infested paddy rice and the rearing substrate attracted approximately 80% of the tested parasitoids. Females also exhibited a strong preference for odours emitted by *O. surinamensis* adults and by fourth‐instar larval faeces compared with the air control. Live larvae free of faeces were not attractive, as no significant differences were observed relative to the air control.

When exposed to 1 ng of each volatile compound identified from larval faeces, tested individual females significantly preferred 1‐pentadecene (70%) over the blank (hexane) (Fig. 2). No significant preference was observed for (*E*)‐2‐nonenal, octanoic acid, phenylacetaldehyde, or (*E*)‐2‐octenal at the same concentration compared to the blank (hexane). However, when 1‐pentadecene was tested against 0.75 g of host larval faeces, over 80% of females chose the faeces. This preference persisted even when a smaller amount of faeces (0.1 g) was tested. Only 8% of the tested parasitoid females showed no response.

### 3.3 Behavioural bioassays in flight cages

Analysis of cumulative captures over 24 h using the AUC revealed significant differences in parasitoid attraction among treatments (*F*₄,₄₀ = 21.08, *P* < 0.001). All treatments were significantly more attractive than the control, while no significant differences were observed among the attractants themselves. The control treatment had the lowest AUC (31.3 ± 3.2), indicating minimal attraction, whereas faeces + paddy rice treatment exhibited the highest AUC (85.6 ± 5.2), followed by 1‐pentadecene (77.6 ± 5.8), faeces (74.1 ± 4.0), and 1‐pentadecene + paddy rice (71.7 ± 4.5).

**Figure 3:**
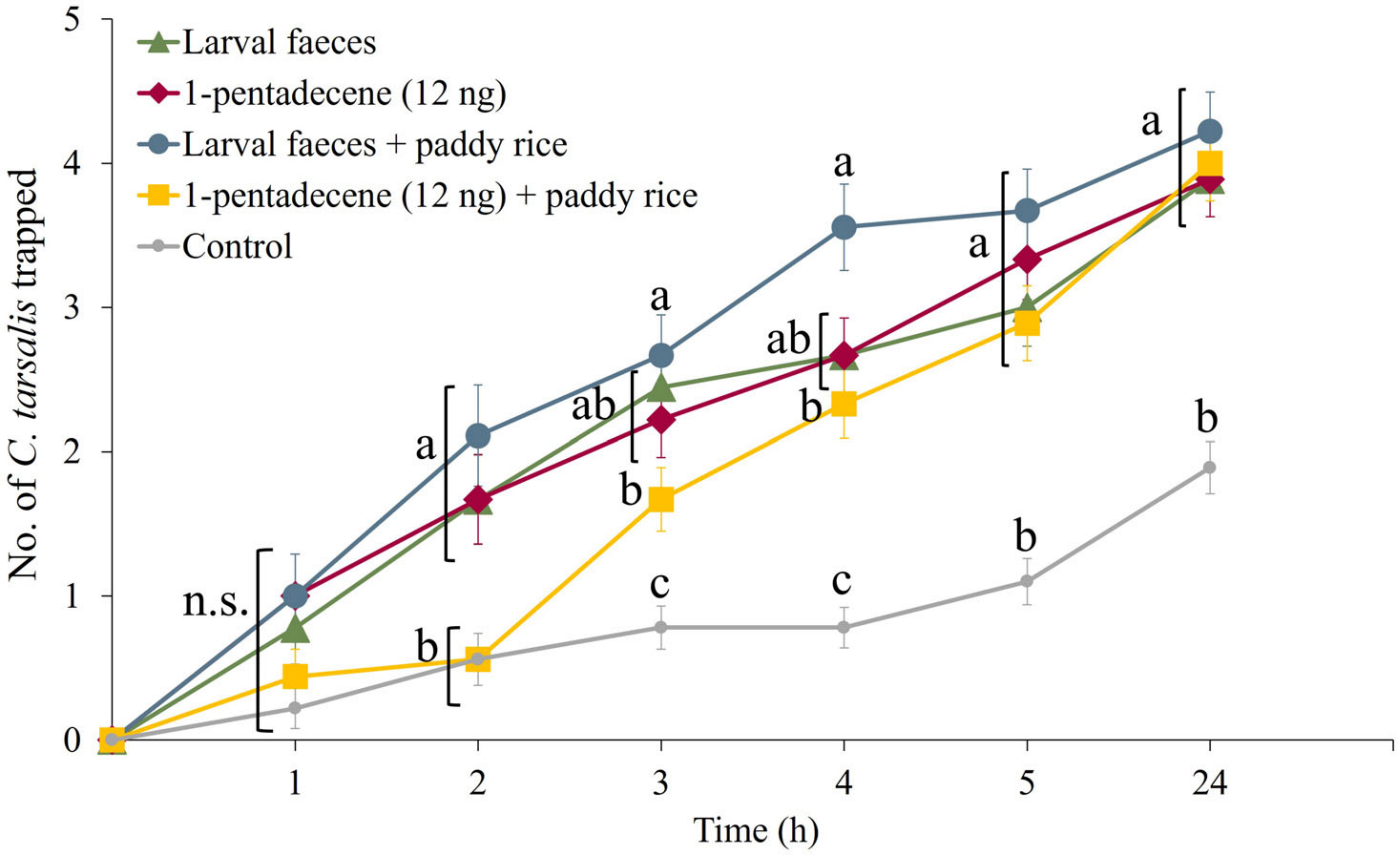
Cumulative number (mean ± standard error) of *C. tarsalis* females trapped per hour on a sticky trap in a flight cage behavioural bioassay assessing the attractiveness of different olfactory sources (*N* = 9). Tested olfactory treatments included fourth‐instar larvae host faeces, 1‐pentadecene (12 ng), faeces + paddy rice, and 1‐pentadecene (12 ng) + paddy rice. Control cages contained no olfactory source. Five females of *Cephalonomia tarsalis* were released per cage. Different letters within the same hour indicate significant differences between treatments (Tukey's test, *P* < 0.05).

In all treatments, captures increased progressively over time, with olfactory treatments attracting an increasingly higher number of parasitoids compared to the control (Fig. 3). When comparing the number of *C. tarsalis* in the traps 1 h after releasing the parasitoids, no significant differences were observed between the five treatments (*χ* ^2^ = 9.55, df = 4, *P* = 0.05). After 2 h, the odours of faeces, 1‐pentadecene and faeces + paddy rice attracted more parasitoids than the control and the 1‐pentadecene + paddy rice treatments (*χ* ^2^ = 30.02, df = 4, *P* < 0.001). By 3 and 4 h, the control treatment attracted significantly fewer parasitoids compared to the other olfactory treatments (3 h: *χ* ^2^ = 44.61, df = 4, *P* < 0.001; 4 h: *χ* ^2^ = 79.49, df = 4, *P* < 0.001), while faeces + paddy rice odour attracted significantly more *C. tarsalis* than the 1‐pentadecene + paddy rice mixture. After 5 h, the number of *C. tarsalis* trapped in the control treatment remained lower than in the other four treatments (*χ* ^2^ = 71.25, df = 4, *P* < 0.001). No significant differences were found among these four treatments in which an average of three *C. tarsalis* were captured throughout the experiment at that time. After 24 h, captures in the control were less than two individuals, significantly lower than the captures in the other four treatments, which were around four individuals (*χ* ^2^ = 66.98, df = 4, *P* < 0.001). When analysing the capture rate of *C. tarsalis* per hour according to the olfactory treatment and time, captures were not significantly affected by the time since exposure (treatment: *χ* ^2^ = 17.59, df = 4, *P* < 0.001; time: *χ* ^2^ = 7.32, df = 4, *P* = 0.120). Therefore, the ratio of captures remained constant during the experiment and was significantly lower in the control treatment.

## 4 DISCUSSION

The present study provides novel insights into the role of host‐specific odours in the searching behaviour of *C. tarsalis* towards its host *O. surinamensis*, highlighting their relevance for understanding and potentially improving the use of this parasitoid in biological control programs.

The chemical analysis of this study allowed the identification of key VOCs involved in the parasitoids' response to host‐associated odours. Some of the compounds detected in *O. surinamensis* larval faeces have also been reported in other coleopteran species. For instance, (*E*)‐2‐nonenal, octanoic acid, decanal, 6,10‐dimethyl‐2‐undecanone, and 1‐pentadecene were identified in the faeces of fourth‐instar larvae of *Tribolium castaneum* ^23^ (Herbst) (Coleoptera: Tenebrionidae). Likewise, larval faeces of *Rhyzopertha dominica* (F.) (Coleoptera: Bostrichidae) were found to contain decane, 1‐decanol, methyl decanoate, 1‐pentadecene, and 1‐tetradecanol.^22^

The olfactometer bioassays demonstrated that uninfested paddy rice and the substrate used for rearing (brown rice) were highly attractive to *C. tarsalis*. This is consistent with previous studies which showed that odour sources associated with host food play an important role in the host‐habitat location of parasitoids.^15^, ^23^, ^26^, ^27^ Although these findings were obtained in different species, they support the broader relevance of such chemical cues, placing our results within a wider functional and applied context. In addition, it is known that adults of *O. surinamensis* also may influence parasitoid behaviour, likely due to the presence of an aggregation pheromone known to guide *C. tarsalis* females.^28^, ^29^

As previously described, our results confirmed the attraction of *C. tarsalis* females to the faeces of *O. surinamensis* fourth‐instar larvae.^15^ Among the VOCs detected exclusively in larval faeces, (*E*)‐2‐nonenal showed a tendency towards eliciting a positive response, while only 1‐pentadecene was significantly attractive to the parasitoid in the olfactometer assays when tested at a dose of 1 ng. This dose corresponds approximately to the amount of 1‐pentadecene present in 60 mg of fresh larval faeces, equivalent to the daily production of around 700 larvae under our experimental conditions (see Table 2). 1‐pentadecene, a long‐chain alkene, is commonly found in insect cuticular hydrocarbons and larval faeces, being associated with metabolic processes associated with feeding and cuticle synthesis. Moreover, it has been reported as a semiochemical involved in intraspecific communication in *Tribolium* spp.^24^, ^30^, ^31^ and in host–parasitoid interactions.^23^

In the flight cage experiment, the parasitoid's response to 1‐pentadecene was similar to that to larval faeces when tested individually, confirming that this hydrocarbon plays an important role in the host‐searching behaviour of *C. tarsalis*. However, when offered in combination with paddy rice, the reduced attraction during the first few hours was likely due to elevated local concentrations of 1‐pentadecene emitted by the paddy rice itself.^22^ This may have temporarily impaired orientation or induced a weak repellent effect^24^ that diminished as the odour dissipated. Further experiments assessing the effect of paddy rice alone in the future would help to better interpret these responses. After 5 h, the source was as attractive as larval faeces, demonstrating that even under more realistic conditions with grain present, 1‐pentadecene remains a key cue for host location in *C. tarsalis*. 1‐pentadecene has also been shown to mediate and improve the host‐location success by the parasitoid *H. sylvanidis* in a similar cage experiment.^10^ In that study, however, 1‐pentadecene was tested in combination with (*E*)‐2‐nonenal, as this additional compound also elicited antennal responses in electroantennogram (EAG) and attracted the parasitoid. Together with our findings, this suggests that 1‐pentadecene alone does not act as a host‐specific cue but rather serves as a general signal that parasitoids integrate with other compounds within a complex odour blend. The involvement of additional VOCs and their combined perception may therefore enhance the parasitoid's ability to efficiently detect and select suitable hosts. Although not examined in the present study, other compounds detected in the faeces may also contribute to host location, either individually or in combination with other volatiles. Their potential contribution remains to be explored and could provide further insight into the complexity of the olfactory cues involved.

The host‐location capacity of *C. tarsalis* has mainly been assessed in medium‐scale assays, where parasitoids respond to naturally emitted odours, such as larval faeces or infested grain, rather than isolated compounds. In static‐air boxes, females reached *O. surinamensis* larval faeces up to 70 cm away,^32^ although in the absence of air movement, detection was limited to just 4 cm. Airflow, such as that generated by convection, improves odour dispersion, allowing parasitoids to locate and parasitize hosts, even at depths of 150 cm in a vertical column of paddy rice.^33^ At larger scales, such as in warehouses, host‐location has not yet been tested in *C. tarsalis*, but studies on related species indicate that volatile cues can operate over larger distances in storage environments.^34^, ^35^

## 5 CONCLUSION

Our findings contribute to a better understanding of the chemical ecology of *C. tarsalis* and provide practical insights for improving its performance as a biological control agent. Identifying key volatile compounds and understanding their synergistic effects could enable the development of strategies to optimise host location and increase pest suppression in storage facilities. The application of semiochemicals could help guiding parasitoids to infested areas, inciting the host‐searching behaviour and thus enhancing the host‐location success and parasitisation rates. Further research is needed to assess the use of host‐associated kairomones as tools to improve the biological control of stored product pests and to determine how the addition of such specific cues influences the ability of parasitoids to locate their hosts within storage environments.

## Data Availability

The data that support the findings of this study are available from the corresponding author upon reasonable request.
